# Thyroid gland invasion in advanced squamous cell carcinoma of the larynx and hypopharynx^☆^^☆☆^

**DOI:** 10.1016/j.bjorl.2016.03.019

**Published:** 2016-05-07

**Authors:** João Mangussi-Gomes, Fernando Danelon-Leonhardt, Guilherme Figner Moussalem, Nicolas Galat Ahumada, Cleydson Lucena Oliveira, Flávio Carneiro Hojaij

**Affiliations:** a Universidade Federal de São Paulo, Departamento de Otorrinolaringologia Cirurgia de Cabeça e Pescoço, São Paulo, SP, Brazil; b Universidade de São Paulo, Laboratório de Pesquisas Clínicas – LIM-32, São Paulo, SP, Brazil

**Keywords:** Squamous cell carcinoma, Larynx, Hypopharynx, Laryngectomy, Pharyngectomy, Thyroidectomy, Carcinoma espinocelular, Laringe, Hipofaringe, Laringectomia, Faringectomia, Tireoidectomia

## Abstract

**Introduction:**

Squamous cell carcinoma of the larynx and hypopharynx has the potential to invade the thyroid gland. Despite this risk, the proposition of either partial or total thyroidectomy as part of the surgical treatment of all such cases remains controversial.

**Objectives:**

To evaluate the frequency of invasion of the thyroid gland in patients with advanced laryngeal or hypopharyngeal squamous cell carcinoma submitted to total laryngectomy or pharyngolaryngectomy and thyroidectomy; to determine whether clinic-pathological characteristics can predict glandular involvement.

**Methods:**

A retrospective case series with chart review, from January 1998 to July 2013, was undertaken in a tertiary care university medical center. An inception cohort of 83 patients with larynx/hypopharynx squamous cell carcinoma was considered. All patients had advanced stage disease (clinically T3–T4) and underwent total laryngectomy or total pharyngolaryngectomy in association with thyroidectomy. Adjuvant therapy was indicated when tumor or neck conditions required. Frequency of thyroid cartilage invasion was calculated; univariate and multivariate analysis of demographic, clinical and pathological characteristics associated with cartilage invasion were performed.

**Results:**

The overall frequency of invasion of the thyroid gland was 18.1%. Glandular involvement was associated with invasion of the following structures: anterior commissure (odds ratio = 5.13; 95% confidence interval 1.07–24.5), subglottis (odds ratio = 12.44; 95% confidence interval 1.55–100.00) and cricoid cartilage (odds ratio = 15.95; 95% confidence interval 4.23–60.11).

**Conclusions:**

Invasion of the thyroid gland is uncommon in the context of laryngopharyngeal squamous cell carcinoma. Clinical and pathological features such as invasion of the anterior commissure, subglottis and cricoid cartilage are more associated with glandular invasion.

## Resumo

**Introdução:**

O carcinoma espinocelular de laringe e hipofaringe tem potencial para invadir a glândula tireoide. Apesar desse risco, a proposição de tireoidectomia parcial ou total como parte do tratamento cirúrgico de todos esses casos permanece controversa.

**Objetivos:**

Avaliar a frequência de invasão da glândula tireoide em pacientes com carcinoma espinocelular avançado de laringe ou hipofaringe submetidos a laringectomia total ou faringolaringectomia e tireoidectomia; determinar se características clínico-patológicas podem prever o envolvimento glandular.

**Método:**

Uma série de casos retrospectivos com revisão de prontuários, entre janeiro de 1998 e julho de 2013, foi feita em um centro médico universitário de cuidados terciários. Uma coorte inicial de 83 pacientes com carcinoma espinocelular de laringe/hipofaringe foi considerada. Todos os pacientes tinham doença em estágio avançado (clinicamente T3-T4) e foram submetidos a laringectomia total ou faringolaringectomia em associação com tireoidectomia. Foi indicada terapia adjuvante quando o tumor ou as condições do pescoço exigiram. A frequência de invasão de cartilagem da tireoide foi calculada; análises univariada e multivariada das características demográficas, clínicas e patológicas associadas à invasão de cartilagem foram realizadas.

**Resultados:**

A frequência global de invasão da glândula tireoide foi de 18,1%. O envolvimento glandular foi associado a invasão das seguintes estruturas: comissura anterior (*odds ratio* = 5,13; intervalo de confiança 95% 1,07-24,5), subglote (*odds ratio* = 12,44; intervalo de confiança 95% 1,55-100,00) e cartilagem cricoide (*odds ratio* = 15,95; intervalo de confiança 95% 4,23-60,11).

**Conclusões:**

A invasão da glândula tireoide é rara no contexto de carcinoma espinocelular laringofaríngeo. As características clínicas e patológicas, como a invasão da comissura anterior, subglote e cartilagem cricoide, estão mais associadas a invasão glandular.

## Introduction

Squamous cell carcinoma (SCC) of the larynx and hypopharynx has the potential to invade the thyroid gland. This invasion occurs mainly by direct extension due to the proximity of this gland to the pharyngolaryngeal region.^1^, ^2^, ^3^ Despite this risk, the proposition of either partial or total thyroidectomy as part of the surgical treatment of all such cases remains controversial.^2^, ^3^, ^4^, ^5^, ^6^

The frequency of neoplastic involvement of the thyroid in advanced SCC of the larynx varies in the literature between 1% and 30%.^4^, ^5^, ^7^ According to these figures, thyroid surgery would be unnecessarily performed in approximately 75% of patients.^3^ Just by adding hemithyroidectomy to the surgical treatment of a laryngeal cancer increases the risks of hypothyroidism and hypoparathyroidism to 23–63% and 25–52%, respectively.^8^, ^9^, ^10^

A definition of the clinical and anatomopathological features associated with thyroid involvement would be of great value in such cases. This definition could direct surgical treatment and reduce morbidity without impairing treatment objectives.^6^, ^11^

The aim of this study was to evaluate the frequency of thyroid gland invasion in patients with advanced SCC of the larynx and hypopharynx undergoing total laryngectomy (TL) or total pharyngolaryngectomy (TPL) associated with Hemithyroidectomy (HT) or total thyroidectomy (TT) and to determine whether clinical and pathological features are able to predict thyroid gland involvement.

## Methods

A retrospective cross-sectional historical cohort study was conducted based on the review of charts and reports of the anatomopathological examination of surgical specimens. All patients undergoing TL or TPL in association with HT or TT for SCC of the larynx and hypopharynx in the period from January 1998 to July 2013 were included.

Patients with metastatic disease were excluded, along with patients undergoing partial or rescue surgery after radiotherapy and patients with incomplete record data.

The surgeries were all performed and all patients monitored at a single Head and Neck Surgery service of a reference university hospital. In this service, surgical excision of the thyroid in association with TL or TPL is indicated for patients with SCC of the larynx and hypopharynx when there is evidence of tumor extension into the following regions: subglottis, pyriform sinuses, and extra-laryngeal tissue. HT or TT is performed according to the laterality of the tumor – HT ipsilateral to the tumor for unilateral tumors; TT for tumors with bilateral involvement.

The choice between TL and TPL depends on the primary site and progression of the tumor into adjacent regions and aims to achieve surgical margins free of neoplasia. Advanced tumors restricted to the larynx require TL; laryngeal tumors with pharyngeal extension or hypopharynx tumors, with or without laryngeal extension, are better treated by TPL.

The following clinical and epidemiological data were analyzed for all patients:

Gender, age, current or past history of smoking;
Primary tumor site;
TNM and overall staging, according to American Joint Committee on Cancer – AJCC criteria;^12^
Surgery performed – TL or TPL, with HT or TT.

The following anatomopathological information was also noted:

Largest tumor diameter under macroscopic examination of the surgical specimen;
Presence or absence of invasion by the primary tumor of the pyriform sinuses, anterior commissure, subglottis, thyroid, and cricoid cartilages.
Presence or absence of macro and/or microscopic thyroid gland invasion;
Histological grade of the tumor;
Presence or absence of angiolymphatic and perineural invasion.

Subglottic invasion was considered when there was extension of the tumor or primary involvement by the neoplasm of the region located more than 10 mm below the true vocal folds.

The frequency of macro or microscopic involvement of the thyroid gland was calculated specifically for SCC for the whole group of patients studied. The patients were then divided into two groups: (1) patients with thyroid gland involvement; (2) patients with thyroid free from carcinoma. The two groups were compared for differences with regard to the variables mentioned above.

The study was approved by the institution Ethics Committee. SPSS v.17, Minitab 16, and Excel Office 2010 were used for statistical analysis. The ANOVA and the two-tailed Fisher's exact tests were applied to analyze quantitative and qualitative variables, respectively. The degree of association between involvement of the thyroid gland and other variables was analyzed by calculating the odds ratio (OR) and its 95% confidence interval (95% CI). Values of *p* < 0.05 were interpreted as statistically significant.

## Results

Data from 83 patients were reviewed and analyzed. Seventy-eight (94.0%) patients were male, and 5 (6.0%) were female. The mean patient age (±SD) was 59.5 ± 10.2 years. Seventy-four (89.2%) patients had a current or past history of smoking. The primary tumor site was the larynx in 68 (81.9%) cases and the hypopharynx in 15 (18.1%) patients. The TNM staging of the patients and the surgery performed are shown in Table 1.

**Table 1:** Patients with laryngeal and/or hypopharyngeal SCC submitted to TL or TPL with HT or TT – clinical data.

Variable	*n* (%)
*T staging*^a^	
T 3	26 (31.3%)
T 4a	57 (68.7%)

*N staging*^a^	
N 0	45 (54.2%)
N 1	10 (12.0%)
N 2a	8 (9.6%)
N 2b	7 (8.4%)
N 2c	11 (13.3%)
N 3	2 (2.4%)

*M staging*^a^	
M 0	83 (100%)
M 1	0 (0%)

*Global staging*^a^	
III	15 (18.1%)
IVa	66 (79.5%)
IVb	2 (2.4%)

*Performed surgery*	
TL/TPL + HT	54 (65.1%)
TL/TPL + TT	29 (34.9%)

SCC, squamous cell carcinoma; TL, total laryngectomy; TPL, total pharyngolaryngectomy; HT, hemithyroidectomy; TT, total thyroidectomy; TL/TPL + HT, total laryngectomy or total pharyngolaryngectomy with hemithyroidectomy; TL/TPL + TT, total laryngectomy or total pharyngolaryngectomy with total thyroidectomy.

a TNM and Global tumor staging, according to American Joint Committee on Cancer (AJCC) criteria.^12^

Thyroid gland invasion by SCC was identified in 15 patients. The frequency of glandular involvement was 18.1% (15/83) for all patients. Among the tumors located primarily in the larynx, the frequency of thyroid gland invasion was 19.1% (13/68). Considering only hypopharynx tumors, the frequency of glandular involvement was 13.3% (2/15). Invasion was macroscopic, by direct extension of the tumor, in 13 (86.7%) patients. Invasion was identified only microscopically in 2 (13.3%) cases.

The comparison between the patient groups with and without thyroid involvement revealed that patients with evidence of glandular invasion showed the following characteristics:

Higher rates of invasion of the following structures by the primary tumor:

• Anterior commissure (*p* = 0.039);
• Subglottis (*p* = 0.003);
• Cricoid cartilage (*p* < 0.001).

The primary tumor invaded the pyriform sinus in only two cases of thyroid involvement. These instances were the only cases of hypopharynx tumors in patients with positive thyroid malignancy. Involvement of the pyriform sinus was statistically less frequent in patients with positive thyroid malignancy (*p* = 0.039).

There was no statistically significant difference between the groups regarding gender, age, present and/or past smoking, primary tumor site, T, N, M, and global staging, size of the tumor, surgery, histologic grade, and the presence of angiolymphatic or perineural invasion. A detailed comparison between the groups is shown in Table 2, Table 3, Table 4.

**Table 2:** Comparison between groups of patients with laryngeal and/or hypopharyngeal SCC submitted to TL or TPL with HT or TT – clinic-epidemiological data.

Variable	Thy Pos	Thy Neg	*p*-value
	*n* (%)	*n* (%)	
*Gender*			1.000
Male	14 (93.3%)	64 (94.1%)	
Female	1 (6.7%)	4 (5.9%)	

*Current or past history of smoking*			1.000
Yes	14 (93.3%)	60 (88.2%)	
No	1 (6.7%)	8 (11.8%)	

*Primary site of the tumor*			0.728
Larynx	13 (86.7%)	55 (80.9%)	
Hypopharynx	2 (13.3%)	13 (19.1%)	

*T staging*^a^			0.129
T 3	2 (0%)	24 (35.3%)	
T 4a	13 (100%)	44 (54.7%)	

*N staging*^a^			
N 0	8 (53.3%)	37 (54.4%)	1.000
N 1	3 (20.0%)	7 (10.3%)	0.377
N 2a	1 (6.7%)	7 (10.3%)	1.000
N 2b	1 (6.7%)	6 (8.8%)	1.000
N 2c	1 (6.7%)	10 (14.7%)	0.679
N 3	1 (6.7%)	1 (1.5%)	0.331

*M staging*^a^			1.000
M 0	15 (100%)	68 (100%)	
M 1	0 (0%)	0 (0%)	

*Global staging*^a^			
III	1 (6.7%)	14 (20.6%)	0.2849
IVa	13 (86.7%)	53 (77.9%)	0.7248
IVb	1 (6.7%)	1 (1.5%)	0.331

*Performed surgery*			0.136
TL/TPL + HT	7 (46.7%)	54 (79.4%)	
TL/TPL + TT	8 (53.3%)	24 (21.6%)	

SCC, squamous cell carcinoma; TL, total laryngectomy; TPL, total pharyngolaryngectomy; HT, hemithyroidectomy; TT, total thyroidectomy; TL/TPL + HT, total laryngectomy or total pharyngolaryngectomy with hemithyroidectomy; TL/TPL + TT, total laryngectomy or total pharyngolaryngectomy with total thyroidectomy; Thy Pos, thyroid gland positive for malignancy; Thy Neg, thyroid gland negative for malignancy.

a TNM and Global tumor staging, according to American Joint Committee on Cancer (AJCC) criteria.^12^

**Table 3:** Comparison between groups of patients with laryngeal and/or hypopharyngeal SCC submitted to TL or TPL with HT or TT – age and largest size of the tumor.

Variable	Thy Pos	Thy Neg	*p*-value
*Age (years)*			
Mean ± SD	60.7 ± 11.5	59.2 ± 9.9	0.678

*Largest size of the tumor (cm)*			
Mean ± SD	5.5 ± 2.7	4.3 ± 1.5	0.943

SCC, squamous cell carcinoma; TL, total laryngectomy; TPL, total pharyngolaryngectomy; HT, hemithyroidectomy; TT, total thyroidectomy; Thy Pos, thyroid gland positive for malignancy; Thy Neg, thyroid gland negative for malignancy; SD, standard deviation.

**Table 4:** Comparison between groups of patients with laryngeal and/or hypopharyngeal SCC submitted to TL or TPL with HT or TT – anatomopathological data.

Variable	Thy Pos	Thy Neg	*p*-value
	*n* (%)	*n* (%)	
*Invasion of adjacent structures*			
Pyriform sinus	2 (13.3%)	30 (44.1%)	0.039^a^
Anterior commissure	13 (86.7%)	38 (55.9%)	0.039^a^
Subglottis	14 (93.3%)	36 (52.9%)	0.003^a^
Thyroid cartilage	12 (80.0%)	37 (54.4%)	0.086
Cricoid cartilage	11 (73.3%)	10 (14.7%)	<0.001^a^

*Histological grade (degree of differentiation)*			
Well differentiated	5 (33.3%)	30 (44.1%)	0.568
Moderately well differentiated	7 (46.7%)	27 (39.7%)	0.773
Poorly differentiated	3 (20.0%)	11 (16.2%)	0.711

*Angiolymphatic invasion*			0.766
Yes	6 (40.0%)	23 (33.8%)	
No	9 (60.0%)	45 (66.2%)	

*Perineural invasion*			0.474
Yes	4 (26.7%)	12 (17.6%)	
No	11 (73.3%)	56 (82.4%)	

SCC, squamous cell carcinoma; TL, total laryngectomy; TPL, total pharyngolaryngectomy; HT, hemithyroidectomy; TT, total thyroidectomy; Thy Pos, thyroid gland positive for malignancy; Thy Neg, thyroid gland negative for malignancy; SD, standard deviation.

a Statistically significant value (*p* < 0.05).

When calculating the odds ratio (OR), thyroid gland invaded by neoplasia was positively associated, with statistical significance, with the involvement of the anterior commissure, the subglottis, and the cricoid cartilages by the primary tumor. The strongest association could be observed with cricoid cartilage involvement (OR = 15.95, 95% CI 4.23–60.11). The OR calculated for each variable, with their respective 95% confidence intervals, are shown in Table 5.

**Table 5:** Association between thyroid gland invasion by SCC and anatomopathological data – odds ratio and 95% confidence interval.

Variable	Thy Pos	Thy Neg	OR (95% CI)
**Invasion of adjacent structures**			
*Anterior commissure*			5.13 (1.07–24.5)^a^
Yes	13	38	
No	2	30	

*Subglottis*			12.44 (1.55–100.00)^a^
Yes	14	36	
No	1	32	

*Cricoid cartilage*			15.95 (4.23–60.11)^a^
Yes	11	10	
No	4	58	

OR, odds ratio; 95% CI, 95% confidence interval.

a Statistically significant values (*p* < 0.05).

## Discussion

SCC of the larynx and hypopharynx, as a whole also called the laryngopharyngeal region, has the potential to invade the thyroid gland. This event was uncommon in the cases presented. The thyroid gland was affected by the tumor in 18.1%, 19.1%, and 13.3% of cases of carcinoma of the larynx and hypopharynx, larynx only, and hypopharynx only, respectively. These data are similar to the results reported by other authors, according to whom values range from 1% to 30%.^3^, ^4^, ^5^, ^7^, ^9^, ^13^

Thyroid gland invasion is a poor prognostic factor in the context of laryngopharyngeal SCC.^6^, ^9^, ^14^ Nevertheless, the frequencies found in this study suggest that thyroid surgery would be performed unnecessarily in more than 80% of these cases. It is known that the performance of thyroidectomy, whether total or partial, greatly increases therapeutic morbidity. The occurrence of hypothyroidism and hypoparathyroidism, for example, greatly increases when thyroid is partially or totally removed.^4^, ^8^, ^9^ Lo Galbo et al., for example, showed that of 37 patients who underwent TL with HT in their series, 78.3% developed hypothyroidism within a 5 year follow up period.^10^ Likewise, Mortimore et al. demonstrated that the incidence of hypoparathyroidism was 25% in patients undergoing TL with HT and adjuvant radiotherapy.^15^

For all these reasons, the indications for thyroidectomy as part of the treatment of laryngopharyngeal carcinoma have been much discussed in the literature. The refinement of criteria that are most strongly associated with glandular invasion would be of great value, particularly in the reduction of endocrine complications.^2^, ^3^, ^4^, ^5^, ^6^

In this study no association of thyroid gland involvement was demonstrated with lymph node staging or with the presence of angiolymphatic and/or perineural invasions and the microscopic analysis of tumors. Furthermore, only 2 (13.3%) cases of thyroid tumor invasion were not detected based on macroscopic extension of the tumor. These findings corroborate the theory that glandular invasion by laryngopharyngeal SCC occurs most commonly by contiguity or direct invasion. Invasion occurred through lymphatic dissemination in only a few cases and, much more rarely, hematogenically.^2^, ^5^, ^9^, ^11^

One might imagine that more aggressive and less differentiated tumors would have a higher frequency of thyroid gland invasion. However, in this study, there was no relationship of glandular involvement with the histological grade of the tumor. This finding is consistent with the findings of other authors and may be explained by the fact that less differentiated tumors are not necessarily larger or more expansive.^4^, ^11^

Although pyriform sinus invasion is a classic criterion for performing thyroidectomy as part of the treatment of laryngopharyngeal SCC, this study demonstrated that this feature was more frequent in patients with neoplasia-free thyroids (*p* = 0.039). The only two cases where there was invasion of the pyriform sinus and thyroid gland were represented by the only hypopharynx tumors in the group of tumors with positive thyroid. That is, in these cases, the pyriform sinus was the primary site of the tumor, and thyroid gland invasion occurred due to downward growth of the carcinoma. The findings, which is in accordance with other studies, indicate that the involvement of the pyriform sinus alone does not indicate glandular thyroid involvement.^3^, ^13^

It was demonstrated that cases with thyroid positive for carcinoma were positively associated, with statistical significance, with invasion of the anterior commissure (*p* = 0.039), the subglottic region (*p* = 0.003), and the cricoid cartilage (*p* < 0.001). This association was repeated in calculating the odds ratio for these variables, which was strongest for cricoid cartilage involvement (OR = 15.95, 95% CI 4.23–60.11). Other studies have shown similar associations to those found here.^2^, ^3^, ^5^, ^7^, ^9^, ^16^ These data can be explained by the fact that these structures are anatomically positioned near areas of weakness of the laryngeal framework: the cricothyroid membrane and the paramedian cricothyroid space. It is precisely through these regions that carcinomas spread to extra laryngeal tissues, especially the thyroid gland.^5^, ^7^, ^8^, ^11^, ^14^

However, not all findings mentioned above are considered predictors of thyroid gland invasion by all authors. A meta-analysis conducted by Mendelson et al. showed that only transglottic or subglottic tumors or tumors with subglottic extension >10 mm were associated with invasion of the thyroid gland. Despite the positive association, no statistically significant correlation was identified with invasion of the cricoid and thyroid cartilages.^11^ One criticism that may be made of that study is that few studies were included in its analysis, particularly with regard to the involvement of the laryngeal cartilages.

In a more recent study, Gaillardin et al. compared preoperative findings with the results of a histological study of the thyroid gland. They demonstrated that over 40% of patients with signs of invasion of the cricoid cartilage in computed tomography (CT) scans had glandular involvement by carcinoma. They concluded that this type of examination is essential in defining thyroidectomy indications.^3^ Likewise, it is suggested that the invasion of structures more related to thyroid gland involvement should be evaluated prior to surgery using the available diagnostic methods, such as endoscopy and CT. The decision regarding the performance of thyroidectomy and its extension should be based on these findings.

This study has inherent limitations in that it is a retrospective study, based on the analysis of medical records and anatomopathological study reports. Especially for this reason, it is subject to information bias. Moreover, although the calculations of odds ratios exhibited statistical significance, the confidence intervals were relatively wide. The small number of patients with thyroid involvement in our sample could explain this result. Studies with larger samples and better methodology are needed to better define indications for thyroidectomy in the context of laryngopharyngeal SCC.

## Conclusion

Thyroid gland invasion by neoplasia is uncommon in the context of laryngopharyngeal carcinoma. Tumors that involve the anterior commissure, the subglottis, and especially the cricoid cartilage are more strongly associated with invasion of this gland.

## Conflicts of interest

The authors declare no conflicts of interest.
